# Functional Relationship between Osteogenesis and Angiogenesis in Tissue Regeneration

**DOI:** 10.3390/ijms21093242

**Published:** 2020-05-03

**Authors:** Francesca Diomede, Guya Diletta Marconi, Luigia Fonticoli, Jacopo Pizzicanella, Ilaria Merciaro, Placido Bramanti, Emanuela Mazzon, Oriana Trubiani

**Affiliations:** 1 Department of Medical, Oral and Biotechnological Sciences, University “G. d’Annunzio” Chieti-Pescara, 66100 Chieti, Italy; francesca.diomede@unich.it (F.D.); guya.marconi@unich.it (G.D.M.); luigia.fonticoli@unich.it (L.F.); ilaria.merciaro@unich.it (I.M.); oriana.trubiani@unich.it (O.T.); 2 ASL02 Lanciano-Vasto-Chieti, “Ss. Annunziata” Hospital, 66100 Chieti, Italy; jacopo.pizzicannella@unich.it; 3 IRCCS Centro Neurolesi “Bonino-Pulejo”, 98124 Messina, Italy; placido.bramanti@irccsme.it

**Keywords:** bone regeneration, angiogenesis, osteogenesis, scaffold, mesenchymal stem cells, wound healing

## Abstract

Bone tissue renewal can be outlined as a complicated mechanism centered on the interaction between osteogenic and angiogenic events capable of leading to bone formation and tissue renovation. The achievement or debacle of bone regeneration is focused on the primary role of vascularization occurrence; in particular, the turning point is the opportunity to vascularize the bulk scaffolds, in order to deliver enough nutrients, growth factors, minerals and oxygen for tissue restoration. The optimal scaffolds should ensure the development of vascular networks to warrant a positive suitable microenvironment for tissue engineering and renewal. Vascular Endothelial Growth Factor (VEGF), a main player in angiogenesis, is capable of provoking the migration and proliferation of endothelial cells and indirectly stimulating osteogenesis, through the regulation of the osteogenic growth factors released and through paracrine signaling. For this reason, we concentrated our attention on two principal groups involved in the renewal of bone tissue defects: the cells and the scaffold that should guarantee an effective vascularization process. The application of Mesenchymal Stem Cells (MSCs), an excellent cell source for tissue restoration, evidences a crucial role in tissue engineering and bone development strategies. This review aims to provide an overview of the intimate connection between blood vessels and bone formation that appear during bone regeneration when MSCs, their secretome—Extracellular Vesicles (EVs) and microRNAs (miRNAs) —and bone substitutes are used in combination.

## 1. Introduction

Bone tissue regeneration can be defined as a complex mechanism based on the interaction between osteogenic and angiogenic processes able to drive bone growth and tissue restoration. Blood vessel formation is a necessary part of bone formation, skeletal development and the osseointegration process, playing a key role in growth factor transport to permit cell viability and interaction.

In the past two decades, several scaffolds were developed and tested as biomaterials to repair bone defects. Although autologous grafts remain the gold standard for bone regeneration, different artificial scaffolds were fabricated to repair critical-sized bone defects.

The success or failure of bone regeneration is based on the central role of the vascularization process, in particular the critical point is the possibility to vascularize the bulk scaffolds, in order to provide adequate nutrients, growth factors, minerals and oxygen for tissue regeneration and to transfer waste products from the healing area.

The ideal scaffolds should be designed to be porous, with a large degree of pore interconnectivity to permit the formation of a vascular network that provides a positive microenvironment for tissue engineering and regeneration.

**Figure 1:**
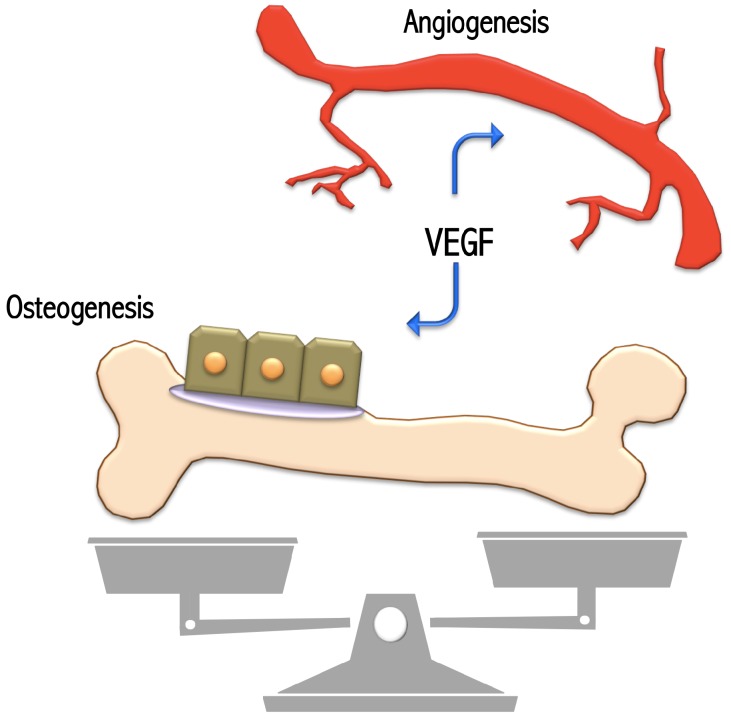
Relevant events regulated by VEGF signal.

Blood vessels exert their function of maintaining cell viability through the perfusion of healing zone during physiological development or bone regeneration. Vascular Endothelial Growth Factor-A (VEGF-A), member of the VEGF family, possesses a main role in angiogenesis. VEGF-A is able to induce the migration and proliferation of endothelial cells and to indirectly stimulate osteogenesis and angiogenesis, regulating the secretion of osteogenic growth factors through paracrine signaling (Figure 1).

Thus, to repair bone tissue defects, we focused our attention on two main categories: the cells and the scaffold that should permit an efficient vascularization process.

Oral tissue can be considered a valid and alternative source of MSCs that possesses the similar features of MSCs derived from bone marrow. For the first time, Dominici et al., in 2006, identified the main features to define a cell population such as MSCs. They wanted to show their capability to adhere to a plastic substrate, possess a fibroblast-like morphology and the capacity to differentiate in vitro into different stromal cell lineages; moreover, they wished to show lack of expression of hematopoietic markers [1]. Using the abovementioned criteria, Gronthos et al. have isolated MSCs from oral tissue derived from adult patients [2,3]. Orally derived MSCs are easy to obtain, isolate and manipulate. They exhibited the capability to adhere to a plastic substrate, a fibroblast-like phenotype, and the ability to differentiate into osteogenic, adipogenic and chondrogenic lineages; furthermore, they displayed positivity for stemness surface markers and negativity for hematopoietic surface molecules [4,5]. 

Orally derived MSCs have been widely studied for use with different biocompatible 3D scaffolds and demonstrated the ability to enhance in vitro and vivo bone formation; in particular, they demonstrated the capacity to induce an endothelial commitment and the promotion of angiogenesis [6].

Scaffold material must provide a three-dimensional structure for cells, mechanical stability, and induce some cellular activity and protein synthesis. In addition, the spatial geometric design should allow endothelial cell migration and the growth of new vessels [7,8]. 

Although many studies are focused on the effect of scaffolds on bone regeneration, few studies have discussed the capacity of the scaffold to provide efficient vascularization and the molecular mechanisms that affect the angiogenesis process, which are necessary to start the bone tissue regeneration.

miRNAs were associated with an active role in osteogenic–angiogenic coupling, these molecular mechanisms that regulate bone angiogenesis are key factors to evaluate and improve the therapeutic strategies in bone regeneration, tissue engineering, and the treatment of bone-related diseases [9].

This review aims to summarize the deep link between blood vessels and bone formation that occurs during bone regeneration when MSCs, their derivatives—extracellular vesicles (EVs) and miRNAs—and bone substitutes are used in combination.

## 2. Bone Regeneration and Stem Cells

At the end of the eighteenth century, it was discovered that cells were organized in specific and distinct layers during a specific stage of embryo development. Based on this observation, during subsequent centuries, many researchers have focused their attention on cell organization at the embryo stage of gastrulation.

Several scientists have focused their research on the tissues derived from the three different germinative layers [10]. Many patterning signals can influence the differentiation process into various organs and tissues. During early embryonic stages, cells are pluripotent and can generate typical cells of the whole organism; however, in the last stages of embryo development, the pluripotency of these cells decrease gradually. These findings suggest that pluripotent factors play a pivotal role in embryo development [11]. As is widely reported in the literature, the development of skeleton and bone tissue is strictly dependent on various morphogenetic growth factors, hormones and other transcriptional regulators able to promote the osteogenic phenotype [12,13]. The high regenerative potential and self-renewing properties of bones underline the main role played by the progenitor cells in bone formation and repair [14]. Osteoblast, osteoclast and osteocyte progenitors are necessary to maintain mineral homeostasis, promote renovation, repair and new bone development. Moreover, bone tissue represents a reservoir of mineral ions and calcium. Angiogenesis, the formation of new blood vessels, plays a pivotal role in bone development and represents a key contributor to the osteogenesis process.

Bone vasculature plays a crucial role in bone construction, remodeling and homeostasis. New blood vessel growth is essential during both primary bone development as well as fracture repair in adults. Both bone repair and bone remodeling implicate the activation and difficult interaction between angiogenic and osteogenic pathways [15].

Inadequate or inappropriate bone vascularity is related to a decreased bone formation. This evidence could be explained by angiogenesis events exhibited in healing processes after injuries [16]. In response to injury, pro angiogenic factors such as thrombin, fibrinogen fragments, thymosin-β4 and growth factors are released in the wound area to promote angiogenesis. In contrast, after injury, angiogenesis inhibitors suppress blood vessel growth and increase the formation of fibrous tissue [17]. One of the most important angiogenic growth factor associated to the healing process is the VEGF, already known as an endothelial cell mitogen, chemotactic agent and inducer of vascular permeability. In addition, VEGF is involved in epithelization and collagen deposition processes as well [18]. Moreover, VEGF plays a decisive role in skeletal development. It has already been reported that VEGF is implicated in many stages of post-natal bone repair and regeneration, such as intramembranous and endochondral ossification. Hence, VEGF regulates the number of inflammatory cells and MSCs involved in repair processes. Therefore, the absence of proper vascularization during bone formation can compromise bone repair processes. Additionally, tissue repair can be altered by poor blood supply but also by the improper juxtaposition of fractured bone ends, the presence of soft tissues or necrotic bone between bone fragments, infections, drugs and systemic disorders. The negative effects of the vascular system delay bone healing [19]. In recent years, in tissue engineering and regenerative medicine have been studied as new approaches to skeletal tissue formation. Despite the high regenerative potentiality and self-renewing ability of bones, complex clinical conditions such as injuries and bone damage require the continuous and huge production of new bone. Unfortunately, many mechanical and functional aspects of bone can decrease during life in relation with age. Recently, in tissue engineering, MSCs have emerged as an auspicious source of cells able to improve osteo-regeneration and avoid conventional surgical techniques [20].

## 3. Stem Cells

Stem cells are unspecialized cells recognized for two characteristics: the ability to differentiate into various cells lineages like skin cells, bone cells, and blood cells, and self-renewal potentiality. Stem cells can be characterized for their origin or their differentiation ability. Based on their origin, stem cells can be divided into embryonic stem cells (ESCs) and non-ESCs. However, based on their capability to differentiate into distinct cell types, these cells can be defined as totipotent, pluripotent, multipotent, oligopotent or unipotent [21]. Totipotent stem cells are typically in the first development stages of zygotes and are the only cells able to divide and differentiate into the cell lineages of the whole organism [22]. Indeed, embryonic stem cells are originated from the Inner Cell Mass (ICM) of embryo blastocysts. This pluripotent group generates ordinary cells in germ layers, but not the extra embryonic structures that exhibit unlimited proliferation capability and pluripotency to differentiate into various cell types originating from the three germ layers; in order to maintain pluripotency, embryonic stem cells can proliferate during the self-renewal process, but they necessarily need to remain undifferentiated [23]. MSCs or multipotent stromal cells exhibited extraordinary potential in animal and human models for the development of novel therapies in regenerative medicine [24,25]. In 2006, the International Society for Cellular Therapy defined parameters to identify MSCs. These cells must adhere under standard culture conditions, must express CD105, CD73, and CD90 and lack the expression of CD45, CD34, CD14 or CD11b. Furthermore, MSCs are characterized by their elevated differentiative capability into osteogenic, chondrogenic, adipogenic, myogenic and neurogenic-like lineages [26,27]. Lastly, MSCs possess multiple paracrine functions that regulate consequences in inflammatory or autoimmune disorders such as muscle-skeletal defects, acute lung injury and osteoarthritis. At the same time, MSCs are acknowledged as primary growth factor secretors and immunomodulatory promoters [28]. Hence, these cells play a crucial part in tissue renewal, repair and healing; moreover, they represent a multi-talent cell source for regenerative therapies [29]. 

## 4. Oral Mesenchymal Stem Cells

**Figure 2:**
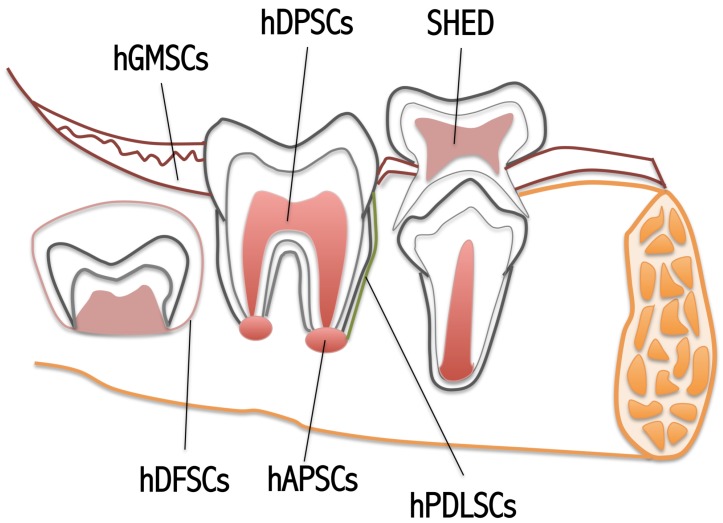
Schematic view of the orally derived MSCs.

The oral cavity is one of the principal sources of MSCs. These cells are derived from neural crests and are a transitory group of embryonic pluripotent stem cells that migrate from the lateral margins of the neural plate toward different tissues. The significance of this reservoir is associated to its easy obtainability and to the considerable numbers of oral tissues from which stem cells can be isolated and characterized [30]. Up to the present time, diverse human oral stem cells have been reported in the literature: human Dental Pulp Stem Cells (hDPSCs), human Exfoliated Deciduous Teeth Stem Cells (SHED), human Periodontal Ligament Stem Cells (hPDLSCs), human Apical Papilla Stem Cells (hAPSCs), human Dental Follicle Stem Cells (hDFSCs) and human Gingival Mesenchymal Stem Cells (hGMSCs) (Figure 2) [31,32]. The last group is characterized by 90% neural crest-derived cells and 10% mesoderm-derived cells [33]. Evaluations among hDPSCs, SHEDs, hPDLSCs and BM-MSCs have established that hDPSCs, SHEDs and hPDLSCs preserve a greater growth potential with respect to BM-MSCs [34]. 

As demonstrated in the literature, hDPSCs, hPDLSCs and BM-MSCs report analogous expression profiles in common cell surface antigens. Human DPSCs, hPDLSCs and human BM-MSCs lack the expression of hematopoietic markers such as CD14, CD18, CD24, CD34 and CD45 and they expressed CD29/integrin beta-1 cell surface receptor, CD44, CD90, CD73, CD105 and CD150 cell surface glycoproteins, CD59 glycoprotein and CD166 transmembrane glycoprotein [35].

Furthermore, in our previous work, we reported that the degree of cell proliferation at the passage 2 (P2) and P15 persist unchanged among hPDLSCs, hDPSCs, and hGMSCs. These data indicate that dental MSCs are very proliferative even at P15. In more detail, hPDLSCs, hDPSCs, and hGMSCs did not evidence any difference in the cell proliferation rate at both P2 and P15 passage [36,37].

## 5. Bone Regeneration 

After bone injuries or fractures, different types of cells and molecules cooperate in order to generate new bone. These events are usually regulated by systemic and local factors that stimulate bone repair. Hence, bone is a tissue with the ability to heal and regenerate itself [38]. Consequently, after a bone fracture, a great number of vessels arrive into the specific damaged site to generate hematomata surrounding the bone defect and allow the secretion of different cytokines involved in the inflammation event. In the neighboring area of the hematoma, VEGF is highly present; this protein promotes the formation of new vessels from surrounding vessels and it also induces the development of an external and internal callus constituted by intermedia cartilage. Soft callus generated in this phase is rapidly replaced by hard callus. This transition starts when intermedia cartilage mineralizes and is progressively replaced by lamellar bone. In the last phase of bone regeneration, the primary lamellar bone is remodeled into secondary bone and restores the regular number of vessels [39]. The great regenerative potential and self-renewing capability of bones is often altered, due to pathologies or the age of patients. For this reason, new bone regenerative treatments are widely required. The purpose of tissue engineering is to overcome these limits and accelerate bone regeneration events. As described, the use of autogenous bone in tissues repair is constantly recommended. Autografts, tissue transplanted from one part of the body to another in the same individual, exhibit osteogenic, osteoconductive and osteoinductive properties and guarantee the absence of transmission diseases. However autologous bones display restricted accessibility, an uncertain quality that can lead to infections and may require additional costs. Inevitably, bone tissue engineering has become one of the most promising branches in regenerative medicine with the aim of overcoming the limits defined by autologous bones. Tissue engineering in bone regeneration often takes advantage of scaffolds and MSCs. Several researchers have developed scaffolds able to reproduce the physical and mechanical nature of autologous tissue and to promote osteoconduction in bone regeneration. Different materials have been already proposed in scaffolds [40]. The principal goal is to reduce cost and hospitalization time in patients that require bone regeneration treatments [41].

## 6. Angiogenesis in Wound Healing

Wound healing is characterized by four phases: hemostasis, inflammatory, proliferative and maturation. In the hemostasis phase, platelets and coagulation factors induce clot and decrease blood loss; in the inflammation event, many inflammatory cells eliminate pathogens and secrete cytokines in the wound area; in the proliferation process, in which the extracellular matrix is reconstructed, the granulation tissues are generated and collagen fiber deposition begins; in the last so-called maturation phase, type III collagen is replaced with type I collagen and the maturation of scar tissue is completed [42]. In fact, after injuries, vasodilation begins with the development of edema and hematoma.

In spite of the prompt augmentation of blood flow to the injured extremity, a phase of necrosis and hypoxia follows, which is a normal part of healing. The necrosis results from mechanical damage to tissue in the peri-fracture region, as well as the loss of nutritional support from the injury to the neighboring blood vessels [43].

Therefore, endothelial cells, angiogenic cytokines, such as Fibroblast Growth Factor (FGF), VEGF, Transforming Growth Factor-Beta (TGF-β) and mast cell tryptase cooperate in a dynamic communication in order to promote new blood vessel formation and injury repair [44]. VEGF-A is the pro-angiogenic factor mostly involved in the healing process. It is remarkable that VEGF-A can increase vascular permeability and contribute to edema formation. In FGF-2, a growth factor member of the FGF family, cardiac ankyrin repeats protein and other factors collaborate with VEGF to promote angiogenesis [45]. The novel production of capillary vessels during repair events is also related to the organization of the extracellular matrix in granulation tissue and in the endothelial basement membrane [46].

As mentioned previously, angiogenesis is recognized as a primary practice in the regeneration and restoration of distinct tissues. The growth of novel blood vessels appears to be fundamental to produce an efficacious cell transplantation amount loaded on numerous scaffolds. Scaffolds are natural or artificial substances that are deliberated as one of the resources for delivering, aligning and keeping cell correlation in support of angiogenesis. Furthermore, the possible function of different scaffold types in vascularization, the application of some approaches such as genetic manipulation, and the conjugation of pro-angiogenic elements could increase angiogenesis prospectively [47].

Recently, bone tissue engineering studies have suggested the using of three-dimensional models in order to promote good and fast healing. Biocompatible materials that support bone growth and development promote the formation of a new vascular network and recruit cells without starting inflammatory events to compose these structures. Different materials with osteoinductive properties are commonly used in the scaffolds, such as calcium phosphates, β-Tricalcium Phosphate (β-TCP), Hydroxyapatite (HA), Polycaprolactone (PCL), Polyglycolic Acid (PGA), Poly-Lactide Acid (PLA), Polylactic Co-Glycolic Acid (PLGA), and bioglass. In addition, biocompatible scaffolds need an appropriate fiber size, porosity and matrix stiffness [48,49]. Scaffolds are often used in combination with stem cells. As mentioned earlier, MSCs represent a virtuous cell source for tissue engineering due to their self-renewal capacity and multi-differentiation potential. Some advantages of their use are: good regeneration ability of damaged tissues, no formation of fibrous structures and low risk of autoimmune rejection due to MSCs immunoregulatory capacity [50]. Dental pulp cells isolated from different oral tissue, such as exfoliated temporal teeth, apical papilla and periodontal ligaments, are frequently employed due to their simple isolation and their capacity to differentiate into cementoblasts and osteoblasts [51]. Human PDLSCs, for example, can differentiate into osteogenic, adipogenic, chondrogenic, and neurogenic cell lineages in vitro. At the same time hPDLSCs can improve bone repair when used in combination with scaffolds, thanks to their high-expansion capability. The immunomodulatory activity of hPDLSCs can be mediated by EVs, paracrine signals containing cytokines, proteins, lipids, and nucleic acids, such as mRNA and microRNAs; furthermore, EVs can regulate osteoblastic differentiation [52]. Human GMSCs are able to differentiate into osteogenic cells. Moreover, this group (hGMSCs) is often associated with biocompatible biomaterials. Furthermore, hGMSCs can release cytokines and growth factors involved in immunomodulatory processes [53].

## 7. Angiogenesis in Bone Regeneration

During the bone regeneration process, different cell lineages interact with each other in order to promote tissue healing. In novel bone development, osteogenic and angiogenic processes are closely connected. The blood vessels of bone tissue can transport minerals and growth factors and, at the same time, represent the physical structures around which bone deposition start. It is largely reported that vascularization plays a crucial part in bone defect repair. Blood vessels, as well as representing indispensable supplement sources, also release paracrine signals that modulate the growth, differentiation and regeneration of different cell types, such as bone cells. The capability of blood vessels to induce VEGF expression, which promotes migration and proliferation of endothelial cells [54] is also notable. As largely reported in the literature, in many animal models, VEGF protein enhanced bone regeneration such as femoral fractures in mice, radius segmental defects in rabbits, and bone-drilling defects in rats [55]. VEGF-A (VEGF), VEGF-B, VEGF-C, VEGF-D, VEGF-E and Placental Growth Factor (PlGF) are included in a big family of homodimeric proteins. The plentiful form in the organism is VEGF-A. Its role is to promote proliferation, migration and activation of endothelial cells, stimulate osteogenesis by osteogenic growth factors and, at the same time, improve vessel permeability. Contrarily, VEGF-B is involved in embryonic angiogenesis, VEGF-C and -D in lymphangiogenesis and PlGF is widely produced during pathological angiogenesis [19]. Osteogenesis is strictly connected to angiogenesis and together contributes to physiological bone function. The physiological impairment of bone healing can be caused by alterations in vascular growth. Moreover, VEGF overexpression may cause bone resorption due to excessive osteoclast presence [56].

## 8. MiRNAs Involved in the Angiogenesis and Osteogenesis

MiRNAs exemplify a group of small, 18- to 28-nucleotide-long, noncoding RNA molecules. To date, 940 members of the family have been recognized in humans. Their main function is in the posttranscriptional regulation of protein expression, and their participation was established in normal and in pathological cellular events. MiRNAs can be defined as “multivalent,” with one miRNA capable of targeting multiple genes, consequently regulating the expression of numerous proteins. Earlier works have recommended that miRNAs may conduct essential roles in cardiovascular and neural formation, stem cell differentiation, apoptosis, and tumor miRNA, presenting entirely novel opportunities for increasing stem cell treatment [57].

Stem cells exert definite miRNA expression profiles, which regulate stem cell destiny [58]. The regeneration of osteogenic cells evidences huge medical research importance. Considerable advancements have been made in generating osteogenic cells from adult stem cells. MiRNAs control osteogenic differentiation through targeting significant transcriptional factors and relative pathways during skeletal formation. The ERK-dependent pathway exhibits a crucial part in osteoblast differentiation. It might stimulate the phosphorylation of Runt-Related Transcription Factor 2 (RUNX2), promote Osterix expression, and increase the action of alkaline phosphatase (ALP) [59,60]. The Focal Adhesion Kinase (FAK) is connected with the stimulation of ERK1/2 through extracellular matrix proteins. MiR-138 represses the differentiation of human MSCs into osteoblasts by directly targeting FAK and downstream signaling [61]. MiR-23b promotes the chondrogenic differentiation of hMSCs by putting down Protein Kinase A (PKA) signaling [62]. 

The latest works have established that several miRNAs exploit principal regulators of bone forming genes, incorporating transcription factors and signaling pathway molecules that are necessary during the osteoblastogenesis. In particular, Bone Morphogenic Protein 2 (BMP2) and RUNX2 exert a chief role in bone development and, moreover, they are indispensable for osteoblast differentiation. Current studies stated the significance of the role played by miRNAs in osteoblast differentiation as regulatory factors, and how miRNAs show a fundamental role in bone gene expression during osteoblastogenesis. As reported in the literature, miR-2861 evidences a positive regulatory role in osteoblast differentiation by blocking Homeobox A2 (Hoxa2) and Histone Deacetylase 5 (HDAC), preserving high levels of RUNX2 mRNA and protein. Furthermore, based on the literature, some miRNAs have been established to act as negative regulators of osteoblast differentiation, such as miR-26a and miR-125b [63,64]. Conversely, miR-29b induces osteogenesis through downregulating numerous inhibitors of osteoblast differentiation [65]; additionally, miR-210 encouragingly controls osteogenesis by inhibiting the TGF-β/activin signaling pathway. Lately, the miRNAs (miR-3960 and miR-2861) that represents an autoregulatory loop in osteoblast differentiation have been described [66]. As reported in the literature, several studies indicated that miR-3960 and miR-2861 target Hoxa2 and HDACs, respectively, indirectly increasing the expression of RUNX2 and promoting osteoblast differentiation. RUNX2 binds to the promoter and activates the transcription of the miR-3960/miR-2861 cluster. Moreover, the silencing of miR-2861 expression resulted in a loss of bone density in mice, supporting the positive regulatory role of miR-2861 in bone formation. MiR-2861 exhibits an optimistic role in regulating osteoblast differentiation and indirectly augments the expression of RUNX2 [67]. In addition, as largely described in the literature, miRNAs are also implicated in stem-cell functions, such as differentiation, by controlling the post-transcriptional process and exhibiting a central role in transduction angiogenic signals. Actually, vascularization is an essential event during osteogenesis and bone regeneration, emphasizing the important role of VEGF during bone repair (Table 1).

Hence, synchronized coupling between osteogenesis and angiogenesis is important and prolonged. The precise interplay of these two primary events is vital during times of rapid bone growth or fracture restoration in adults. Further to VEGF, the new outcomes of the significant regulatory and transforming functions of microRNAs also encourage this crucial mechanism [9]. 

**Figure 3:**
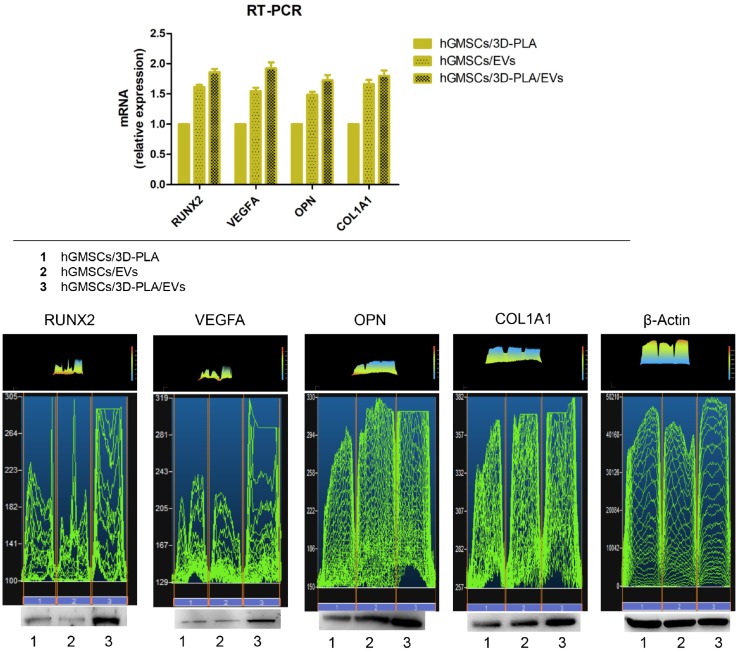
RUNX2 and VEGF expression. RT-PCR showed the different mRNA expression in hGMSCs/3D-PLA, hGMSCs/EVs and hGMSCs/3D-PLA/EVs. Western blot analysis of protein expression: RUNX2, VEGFA, OPN and COL1A1. * *p* < 0.05. Original figure published in: Pizzicannella J. et al [53].

New studies reveal that miR-210 plays a critical role in cell survival and angiogenesis [68]. Our earlier in vitro studies established the upregulation of miR-2861 and miR-210 in hGMSCs/EVs and a further increase in both miRNA was found in 3D-PLA/hGMSCs/EVs (Figure 3). 

It is already recognized that EVs derived from MSCs contain miR-210 and that this evidences a pro-angiogenic outcome. In the same study, the upregulation of miR-2861 and miR-210 was related to augmented VEGF and RUNX2 expression and osteogenic differentiation. These results previously showed that EVs, due to their miRNA content, evidence a chief role in the osteoangiogenic event. Likewise, during bone formation, miR-2861 evidence a positive regulatory role, targeting Hoxa2 and HDACs, respectively, and indirectly favoring an increase in RUNX2 [53,69].

## 9. Extracellular Vesicles (EVs)

EVs have been recognized as one of the intercellular communication mechanisms. EVs are lipid membrane vesicles released by cells with a strategic role that includes paracrine or autocrine biological effects in tissue metabolism [70]. They are present in biological fluids and contain several different active biomolecules, such as proteins, nucleic acids and metabolites. They could represent an important tool for cell-free therapy in regenerative medicine, promoting different cell and tissue activities, such as cell proliferation and viability, angiogenesis and immune responses [71]. EVs derived from MSCs showed paracrine effects without the direct use of living cells in order to avoid ethical concerns and the limitations in administering living cells. The main advantages in the use of EVs regard their safety and manipulation [30]. The functions of EVs in bone metabolism and bone regeneration have been widely reported in the recent literature [72]. Recently, several biomaterials used in the repair of bone defects were loaded with EVs to ameliorate the reparative process, giving promising results. Our previous studies suggested that biomaterials enriched with human oral mesenchymal stem cells (hOMSCs) and EVs are capable of inducing bone regeneration. In particular, EVs functionalized with polyethylenimine, to ameliorate their interaction with cells, improved the mineralization process and induced an extensive vascular network, which is necessary to start an osseointegration event [48,73].

## 10. Conclusions

In the present review, we take into consideration recent studies exploring the key role of angiogenesis and its regulation during the early steps of the osteogenic process. Although many studies indicated that EVs are capable of inducing osteogenesis and angiogenesis, the specific molecular mechanism remains elusive. Finally, a better understanding of the EVs role is necessary to define the real regulation of angiogenesis before starting osteogenic induction.

## Figures and Tables

**Table 1:** MicroRNAs role in the osteogenesis and angiogenesis.

MiRNAs	Role	Reference
*miR-2861*	Induction of osteoblast differentiation by blocking Hoxa2 and HDAC5 preserving high levels of RUNX2 mRNA and protein.	[67]
*miR-23b*	Promotion of chondrogenic differentiation of hMSCs by putting down PKA signaling.	[62]
*miR-138*	Repression the differentiation of hMSCs into osteoblasts by directly targeting FAK and downstream signaling.	[61]
*miR-3960*	Autoregulatory loop in osteoblast differentiation.	[66]
*miR-29b*	Induction of osteogenesis through down regulating inhibitors of osteoblast differentiation.	[65]
*mir-210*	Role in cell survival and angiogenesis.	[69]
*miR-26a* *miR-125b*	Negative regulators of osteoblast differentiation.	[63]

## Abbreviations

VEGF	Vascular Endothelial Growth Factor
MSCs	Mesenchymal Stem Cells
OMSCs	Oral Mesenchymal Stem Cells
EVs	Extracellular Vesicles
ICM	Inner Cell Mass
BM-MSCs	Bone Marrow Mesenchymal Stem Cells
AD-MSCs	Adipose Tissue Mesenchymal Stem Cells
UCB-MSCs	Umbilical Cord Blood Mesenchymal Stem Cells
PMSCs	Placenta Mesenchymal Stem Cells
hDPSCs	human Dental Pulp Stem Cells
SHED	human Exfoliated Deciduous Teeth Stem Cells
hPDLSCs	human Periodontal Ligament Stem Cells
hAPSCs	human Apical Papilla Stem Cells
hDFSCs	human Dental Follicle Stem Cells
hGMSCs	human Gingival Mesenchymal Stem Cells
P2/P15	passage2/passage15
FGF	Fibroblast Growth Factor
TGF-β	Transforming Growth Factor Beta
β-TCP	β-Tricalcium Phosphate
HA	Hydroxyapatite
PCL	Polycaprolactone
PGA	Polyglycolic Acid
PLA	Poly-(Lactide)
PLGA	Polylactic Co-Glycolic Acid
PlGF	Placental Growth Factor
MiRNA	Micro RNA
RUNX2	Runt-Related Transcription Factor 2
PKA	Protein Kinase A
FAK	Focal Adhesion Kinase
BMP2	Bone Morphogenic Protein 2
HDAC	Histone Deacetylase
Hoxa2	Homeobox A2
